# What can we learn from a Chinese social media used by glaucoma patients?

**DOI:** 10.1186/s12886-023-03208-5

**Published:** 2023-11-20

**Authors:** Junxia Fu, Junrui Yang, Qiuman Li, Danqing Huang, Hongyang Yang, Xiaoling Xie, Huaxin Xu, Mingzhi Zhang, Ce Zheng

**Affiliations:** 1 Department of Ophthalmology, School of Medicine, Xinhua Hospital Affiliated to Shanghai Jiao Tong University, 200092 Shanghai, China; 2 Institute of Hospital Development Strategy, China Hospital Development Institute, Shanghai Jiao Tong University, 200092 Shanghai, China; 3 Joint Shantou International Eye Center of Shantou University and the Chinese University of Hong Kong, Shantou University Medical College, Shantou, Guangdong China; 4 Department of Ophthalmology, The 74th Army Group Hospital, Guangzhou, Guangdong China; 5 Department of Pediatric Cardiology, Guangzhou Women and Children’s Medical Center, Guangzhou, Guangdong China; 6 The Faculty of Science, University of Technology Sydney, Sydney, Australia

**Keywords:** Glaucoma, Social media, Word cloud analysis

## Abstract

**Purpose:**

Our study aims to discuss glaucoma patients’ needs and Internet habits using big data analysis and Natural Language Processing (NLP) based on deep learning (DL).

**Methods:**

In this retrospective study, we used web crawler technology to crawl glaucoma-related topic posts from the glaucoma bar of Baidu Tieba, China. According to the contents of topic posts, we classified them into posts with seeking medical advice and without seeking medical advice (social support, expressing emotions, sharing knowledge, and others). Word Cloud and frequency statistics were used to analyze the contents and visualize the keywords of topic posts. Two DL models, Bidirectional Long Short-Term Memory (Bi-LSTM) and Bidirectional Encoder Representations from Transformers (BERT), were trained to identify the posts seeking medical advice. The evaluation matrices included: accuracy, F1 value, and the area under the ROC curve (AUC).

**Results:**

A total of 10,892 topic posts were included, among them, most were seeking medical advice (N = 7071, 64.91%), and seeking advice regarding symptoms or examination (N = 4913, 45.11%) dominated the majority. The following were searching for social support (N = 2362, 21.69%), expressing emotions (N = 497, 4.56%), and sharing knowledge (N = 527, 4.84%) in sequence. The word cloud analysis results showed that ocular pressure, visual field, examination, and operation were the most frequent words. The accuracy, F1 score, and AUC were 0.891, 0.891, and 0.931 for the BERT model, 0.82, 0.821, and 0.890 for the Bi-LSTM model.

**Conclusion:**

Social media can help enhance the patient-doctor relationship by providing patients’ concerns and cognition about glaucoma in China. NLP can be a powerful tool to reflect patients’ focus on diseases. DL models performed well in classifying Chinese medical-related texts, which could play an important role in public health monitoring.

## Introduction

As one of the most common chronic eye diseases, glaucoma is the second leading cause of blindness worldwide, and early detection, early diagnosis, and early treatment are essential to save vision [1]. From 1990 to 2015, the number of glaucoma patients showed an increasing trend, and approximately 25 million Chinese people will live with glaucoma by 2050, which accounts for 1.8% of the total population [1]. And many studies have explored the relationship between glaucoma, anxiety, and depression [2–10], although there is no actual evidence for this, it is reasonable to assume that glaucoma patients have a higher prevalence of psychological disorders. The treatment also affects patients’ life quality. Local or systemic side effects, difficult administration, and complex medication regimens reduce patients’ satisfaction with therapy[11]. On the other hand, patients satisfied with treatment outcomes are more likely to insist on treatment and continue to seek and receive medical services [12, 13]. A good relationship helps to relieve anxiety and improve compliance. Due to a lack of quality medical service resources in most part of China, most doctors care more about disease itself not the patients overall. Doctors are accustomed to assessing efficacy using intraocular pressure (IOP) and visual field (VF). However, from the patients’ perspective, life-related issues, such as reading, taking stairs, and recognizing objects, are more caring [14]. These differences undoubtedly increase the difficulty of doctor-patient communication.

With the development of information technology, the Internet breaks through the limitations of space and time distance, patients tend to express their feelings and experience via social media. As a tool for people to share their opinions, social media has a large number of users. Notably, it is estimated that 72% of internet users read or watch online health information, and 26% post or share their personal health information [15]. Social media has an unprecedented sample size, and as a public health platform, it can provide timely information, including disease detection, health communication, and sentiment analysis. Up to now, several studies have analyzed social media [16, 17], search engine queries [18, 19], and Wikipedia usage to assess and monitor the health status of a population. As one of the largest Chinese social media, Baidu Tieba is an online community with games, entertainment, technology and other themes, and has a large number of friends and fans, you can find topics you are interested in, share your life, hobbies, talents, etc., and participate in various activities and discussions, and it allows users to search or create “bars” (like subreddits) based on specific keywords [20], and users can post their situations and suffer on bars. However, there was no studies explored the demand of the glaucoma patients in China now. To better serve the patients and ease tensions between doctors and patients, it is important to study the needs of glaucoma patients through online communities or social media.

Artificial intelligence (AI), made up of different fields, such as machine learning and computer vision, has developed rapidly in recent years. As a component of machine learning, deep learning (DL) has a broad application prospect in ophthalmology [21]. A combination of social media and DL for public health research is a thriving area. Therefore, we collected topic posts from the Baidu Tieba glaucoma bar to explore the needs of the glaucoma population through Nature Language Processing (NLP). We further developed and validated two DL models to automatically evaluate the ability to recognize medically-related Chinese texts on Chinese social media.

## Materials and methods

We retrospectively collected de-identified social media data from the glaucoma bar of Baidu Tieba from July 16, 2016, to October 11, 2021. According to social media’s privacy policy, de-identified data can be used without authorization from data subjects if that data were used for academic research [20]. The informed consent was exempt, and approval from the institutional review board of both Xinhua Hospital Affiliated to Shanghai Jiao Tong University School of Medicine and Joint Shantou International Eye Center of Shantou University and Chinese University of Hongkong was obtained (identifier, EC 20,190,911 (4) -P11 and XHEC-D-2022-230, respectively). All methods were performed in accordance with the relevant guidelines and regulations.

### Data collection and annotation

The raw datasets, including the posting time, topic, and content, were collected from the glaucoma bar of Baidu Tieba (https://tieba.baidu.com/p/5515066483), using a web crawler program (complied by Python3.7) and JetBrains (PyCharm Community Edition in 2018.3.2). As the world’s largest Chinese community, Baidu Tieba allows users to search or create “bars” (similar to subreddits) for different keywords, publish or reply to posts, and get information or participate in post discussions. The glaucoma-related bar was created in 2004 and currently has more than 17,346 members.

The following posts were excluded, including (1) duplicate posts (same contents that the same user posted on the same day), (2) posts that contain personal privacy, such as portraits, ID cards, specific residential addresses, etc., and (3) posts without contents. According to the nature of the post, we further classified posts into five broad categories refer to previous studies [22]: (1) seeking medical advice, including drug-related, physical signs or examination-related, surgery-related, and others; (2) social support related, including seeking social help or providing social assistance; (3) expressing emotions, either positive or negative emotions; (4) sharing knowledge; (5) others.

Test data was randomly extracted using python random module. The remaining 10,892 posts were divided into training set and verification set according to 7:3 ratio. All posts were independently reviewed by four residents (JRY, QY, FB with two years of residency training in ophthalmology, and QML with two years of residency training in pediatrics). If annotations contradicted each other, a second annotation was performed by a 5th proofreader (CZ, glaucoma specialist), who was blinded to the previous annotations. To determine the consistency among the individuals, one senior ophthalmologist and two students with non-medical backgrounds classified 500 random posts, and the results were analyzed using the Kappa value.

### National language processing (NLP) and development of deep learning (DL) algorithms

For data preprocessing, we first used the N-gram model (N = 3) to correct Chinese spelling errors [23]. An N-gram model is a probabilistic language model for predicting the probability of a sequence of words using the Markov model. We then tokenized the original corpus using an open-source Python library (Jieba, version 0.42.1). Jieba used a detailed Chinese word library to determine the correlation probability of each Chinese word and automatically divided the texts into word sequences. Third, we ignored common Chinese punctuation marks (e.g., “because”, “so”, “and”, “thus” etc.) or stopwords (e.g., “you”, “it”, “she”, “he” etc.) using stopwords list (https://github.com/baipengyan/Chinese-StopWords, provided in the public domain by Baidu) that were not related to the content of the text. Finally, we also removed the words “glaucoma”, “doctor”, and “hospital” to avoid having many topic words directly associated with glaucoma disease. All texts with corresponding labels were collated in Excel for further analysis.

We used word cloud (Word Cloud 1.6.0 in Python3.7) to visualize and highlight the Chinese words with high frequency in the text. Word cloud analysis is an algorithm that can filter a large amount of text information and highlight the key information of the text [24]. As most of the words in posts were in Chinese, we further used Python Translation to translate keywords into English.

In the current study, we adopted two state-of-the-art DL models for automated detection of posts seeking medical advice from other posts (seeking social support, expressing emotions, sharing knowledge, and others). The 2 DL models were (1) the Bi-LSTM model and (2) Bidirectional Encoder Representations from Transformers (BERT) model. Details of Bi-LSTM and BERT models have been described previously. In brief, Bi-LSTM is derived from a Recurrent Neural Network, which typically has the ability to process and predict important events with very long intervals and delays in time series [25]. Bi-LSTMs consist of two separate bidirectional hidden layers that feed forward to the same output layer and maintain contextual features from both past and future states while avoiding the vanishing or exploding gradients problem[26]. BERT is the newest deep representation-learning model [27]. It uses bidirectional transformers to generate word representations, which are jointly conditioned on both the left and right context in all layers. BERT has improved many NLP tasks [28], including question-answering and natural language inference. To implement Bi-LSTM and BERT models, we first obtain Chinese word2vec embeddings through Li et al., 100 + Chinese Word Vectors [29]. Word embedding represents each word in a vector of its surrounding words and can address the sparse entity and word variation issues in social media [30]. The Bi-LSTM and BERT models then used word embedding as the representation of text input. The outputs of the Bi-LSTM were processed to a Softmax classifier, which predicts the categories (seeking medical advice vs. social advice) in the input posts. To implement the BERT model, we adopt the pre-training BERT models using the open-source scripts (the Google AI Research team from the official BERT GitHub repository, github.com/google- research/bert). We used transfer learning and fine-tuned the pre-training BERT model for our specific tasks. All the models in our experiments were trained and tested using Keras API (version 2.2.4) and Keras-Bert API (https://github.com/CyberZHG/keras-bert) with Tensorflow framework (Google, version 2.1.0) as the backend. The computer used in this study was equipped with NVIDIA GTX 1050Ti 4 GB GPU, 24 GB RAM, and AMD Ryzen 3 1300X Quad-Core Processor 3.5 GHz CPU.

### Statistic analysis

SPSS 21.0 (SPSS, Inc, Chicago, IL) was used for data analysis. The categorizing variables were described by rate or percentage, and Cohen’s Kappa coefficient was used to evaluate the consistency among observers. We applied accuracy, specificity, sensibility, F1-score, and Receiver Operating Characteristic curves (ROC) and calculated Area Under Curves (AUC) and 95% confidence interval (95%CI) to evaluate the performance of NLP algorithms.

## Results

**Fig. 1:**
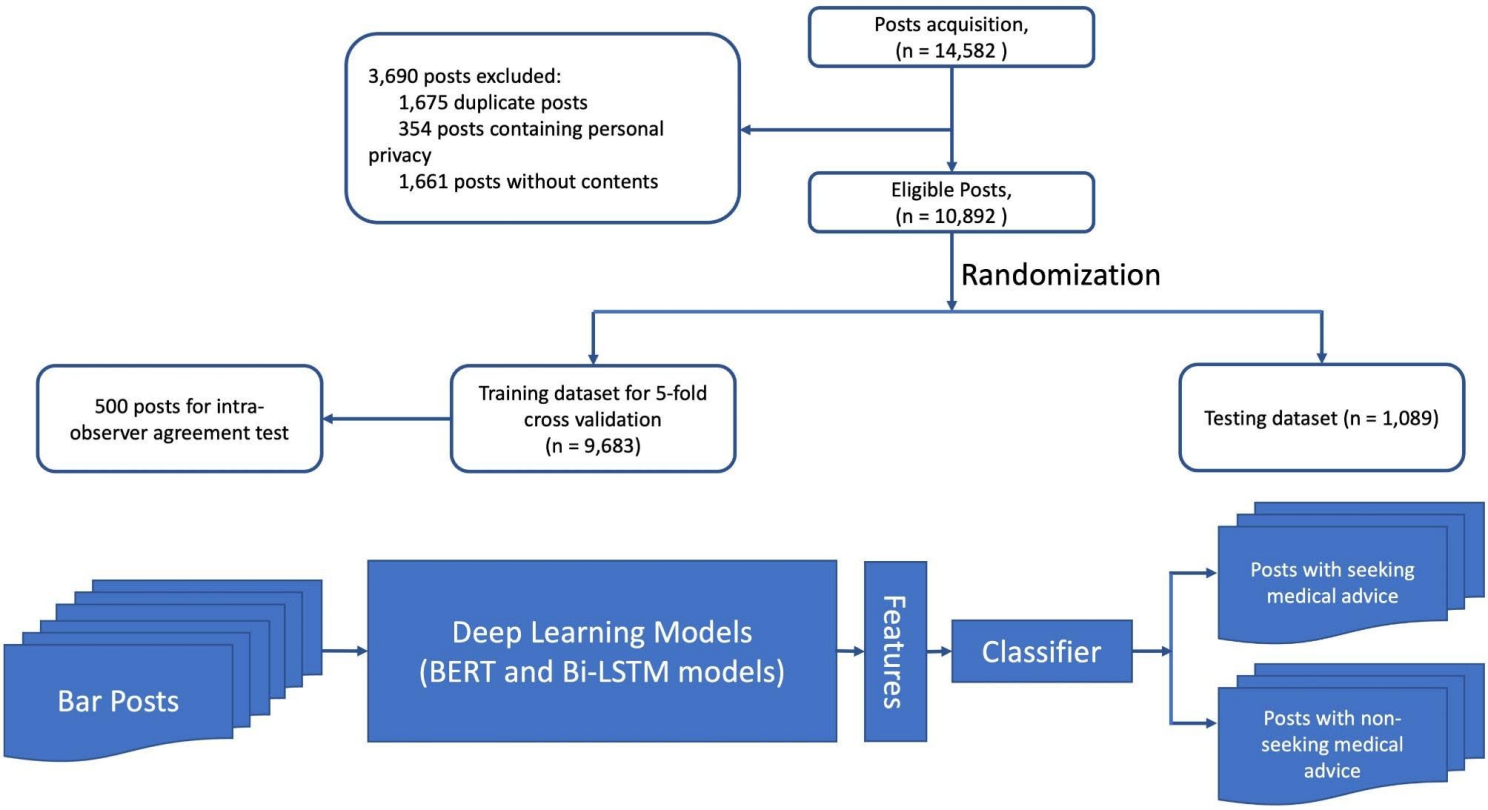
Flowchart illustrating the number of bar posts used to develop, train and test the deep learning algorithm. BERT, Bidirectional Encoder Representations from Transformers; Bi-LSTM, Bidirectional Long Short-Term Memory

**Fig. 2:**
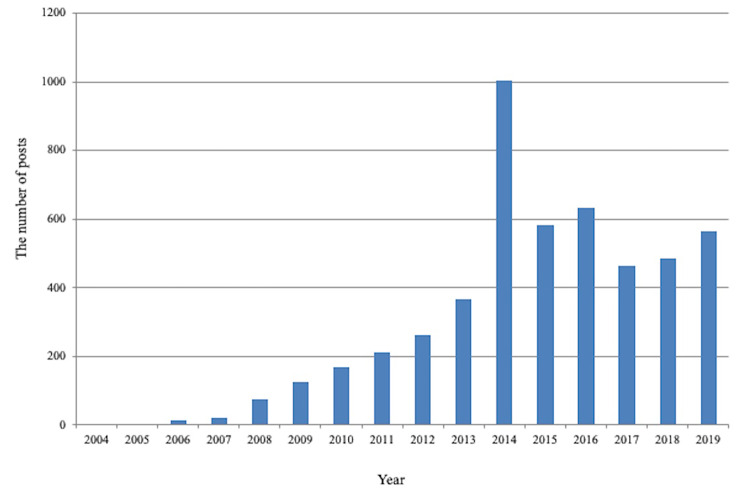
Number of posts about glaucoma in Tieba from 2004 to 2019

14,582 topic posts were collected from Baidu Post Bar between July 16, 2016, to October 11, 2021. Figure 1 shows the flow chart of data crawling and manual classification. Among them, 3,690 (25.3%) topic posts were excluded due to the following reasons: 1,675 (45.4%) duplicate posts, 354 (9.6%) posts containing personal privacy, and 1661 (45.0%) posts without content. After data preprocessing, a corpus of 10,892 posts (66.2%) comprising 309,068 Chinese words was obtained. Finally, training set has about 216,342 tokens, the validation set has about 92,726 tokens, the training set has 7624 posts, and the verification set has 3268 posts. Totally, the number of posts about glaucoma has increased in recent years. (Fig. 2)

We randomly selected 500 topic posts to test intra-observer agreement of annotation of topic posts. Kappa value ranged from 0.643 to 0.778 among five annotators, which was considered acceptable proposed by Landis and Koch [31]. Table 1 showed that the dominant proportion of topic posts in the Glaucoma bar was seeking professional medical advice (N = 7,071, 64.91%), including 4,913 posts (45.11%) seeking advice regarding symptoms or examination. The second proportion of topic posts were posts related to social support (both seeking and providing social support), and 1,355 posts (12.44%) provided social support, among which 678 (50.03%) were classified as hospital advertisements (with a fixed format containing hospitals’ names). Finally, of the 475 posts expressing negative emotions, 226 posts (47.6%) were related to being diagnosed with or suspected glaucoma, followed by expressing negative emotions (n = 83, 17.47%) about prognosis.

**Table 1:** Posting content classified results

Topic	n	%
**Seeking medical advice**		
Drug	982	9
Examination/Symptom	4913	45.11
Surgery	1176	10.8
Total	7071	64.91
**Social support**		
Seeking social support	1007	9.25
Providing social support	1355	12.44
Total	2362	21.69
**Expressing emotions**		
Negative emotion	475	4.36
Positive emotion	22	0.2
Total	497	4.56
**Sharing knowledge**	527	4.84
**Others**	435	4

**Fig. 3:**
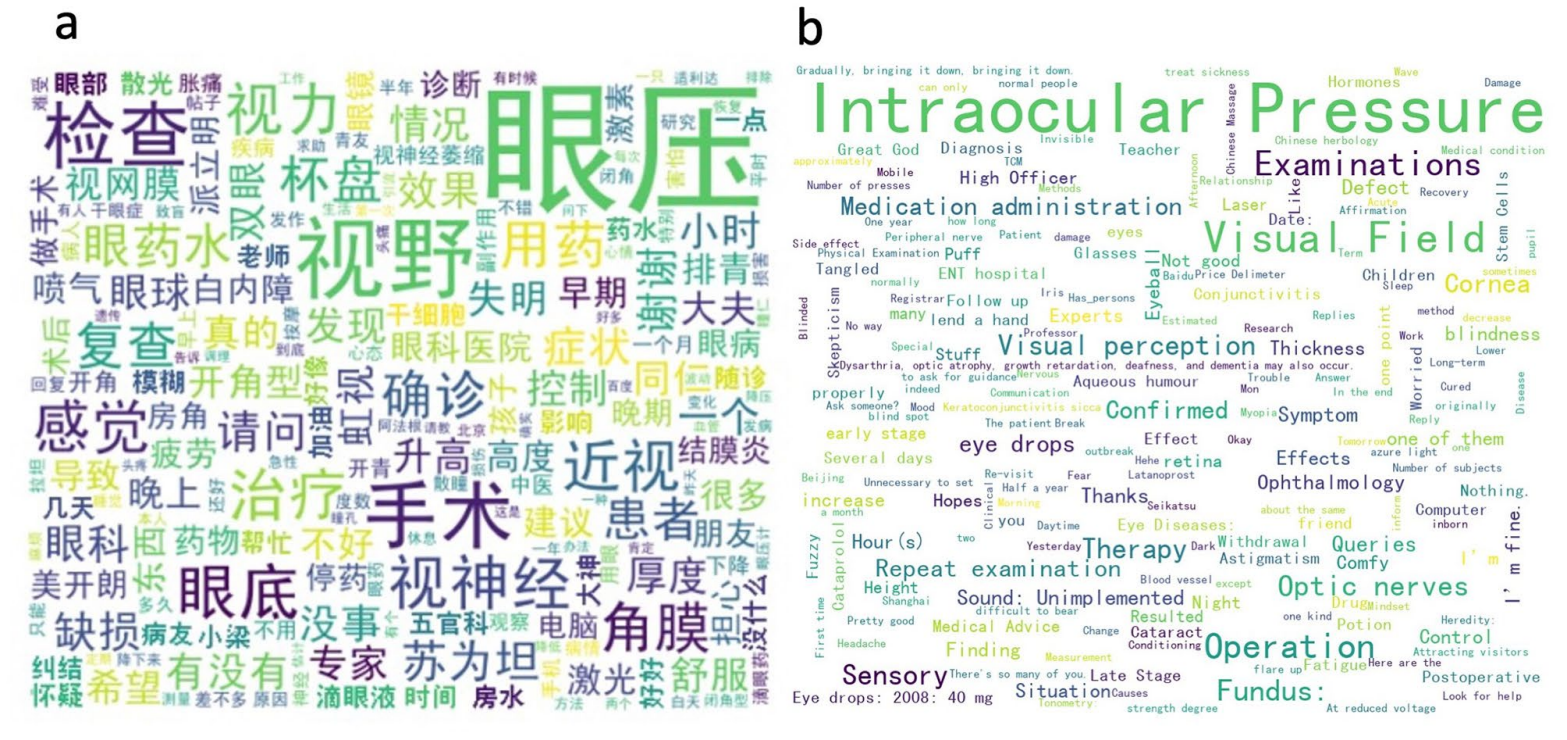
Word cloud reflects the top keywords from glaucoma bar posts in both Chinese (**a**) and English (**b**)

We counted the frequency of each Chinese word in posts and summarized them in a word cloud format. Figure 3a-b shows word clouds of the top keywords from posts in both Chinese and English. The word cloud analysis showed that “intraocular pressure (IOP)” (n = 14,810, 4.55%), “visual field (VF)” (n = 4,686, 1.44%), “examination” (n = 4,258, 1.31%), and “operation” (n = 3010, 0.93%) were the most frequent words in the posts. The drug name “Travoprost” (n = 563, 0.17%) occurred most, followed by “steroid” (n = 477, 0.15%), “Brinzolamide” (n = 302, 0.09%), and “Carteolol” (n = 288, 0.09%). Regarding the symptoms of glaucoma, the most frequent words were “blind” (n = 706, 0.22%), “blurring” (n = 514, 0.16%), “haloes” (n = 509, 0.16%), and “ocular pain” (n = 387, 0.12%) respectively. Negative words such as “worried”(n = 602, 0.19%), “afraid”(n = 460, 0.14%), and “uncomfortable” (n = 365, 0.11%) were also common, while optimistic words were hardly seen.

**Fig. 4:**
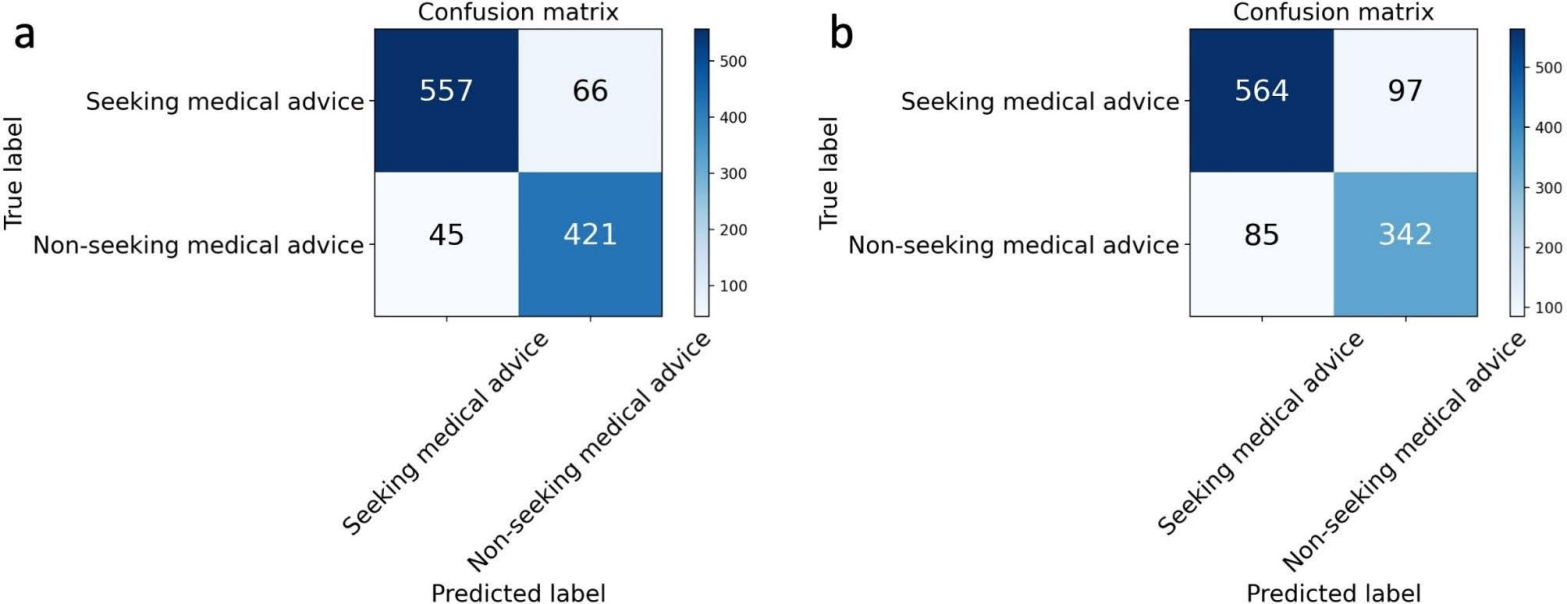
Confusion matrices for BERT (Fig. 3a) and Bi-LSTM mode (Fig. 3b) in the testing dataset. BERT, Bidirectional Encoder Representations from Transformers; Bi-LSTM, Bidirectional Long Short-Term Memory

**Fig. 5:**
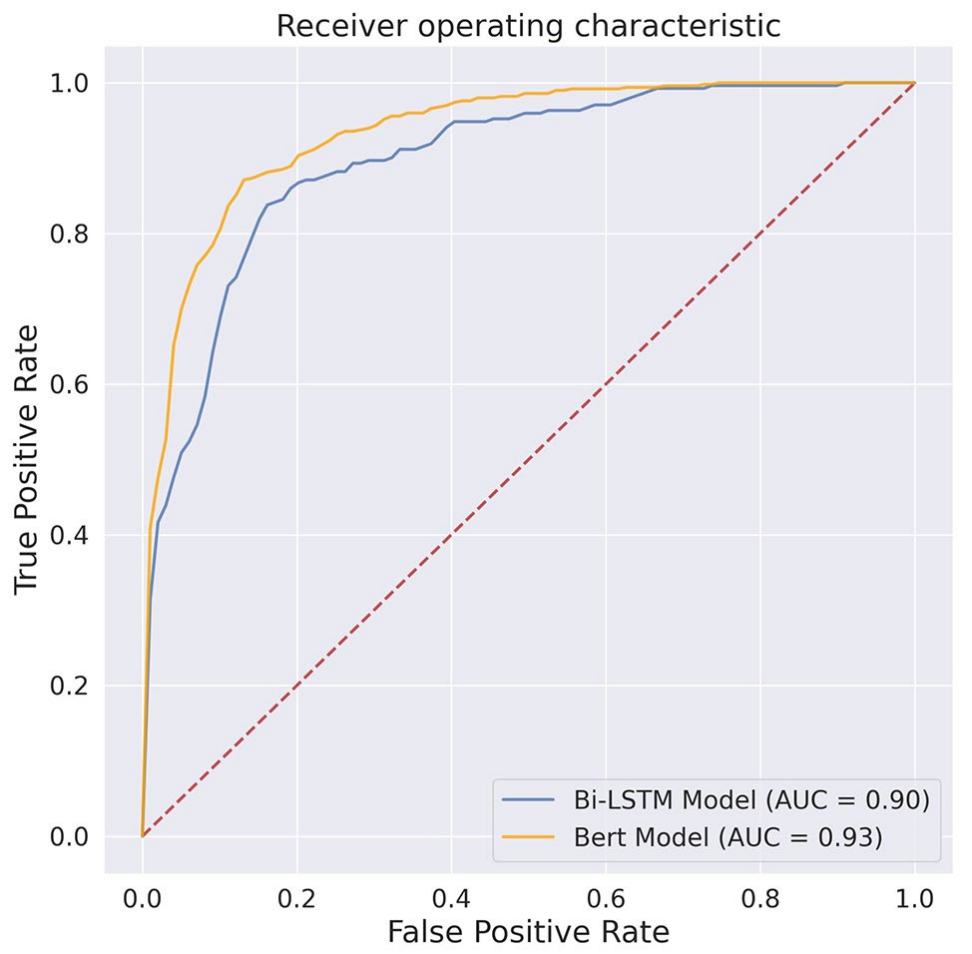
The average AUCs of two DL models tested in the testing dataset. AUC: the area under the receiver operating characteristic curve; DL, deep learning; BERT, Bidirectional Encoder Representations from Transformers; Bi-LSTM, Bidirectional Long Short-Term Memory

For the detection of posts seeking medical advice, the BERT models achieved better performance across all metrics than the Bi-LSTM model (Fig. 4a-b). The accuracy, F1 score and AUC of the BERT model were 0.89 (95%CI 0.87 ~ 0.91), 0.89 (95%CI 0.87 ~ 0.91), and 0.93 (95%CI 0.90 ~ 0.95), respectively. Whereas, for the Bi-LSTM model, the accuracy, the F1 value, and the AUC were 0.82 (95%CI 0.79 ~ 0.84), 0.82 (95%CI 0.80 ~ 0.84), and 0.90 (95%CI 0.87 ~ 0.91), respectively (Fig. 5).

## Discussion

Machine learning and NLP models have been highly topical issues in medicine in recent years and may be considered a new paradigm in medical research. Many studies have confirmed that social media reflects the needs and tendencies of people [32–36], and natural language processing (NLP) shows superior performance in the classification of emotional tendencies, and it also has a good performance in Chinese text. Our study revealed that the DL-based NLP model performs well in classifying Chinese medical-related texts. As a result, Chinese social media was able to reflect people’s concerns and cognition about diseases and the effectiveness of doctor-patient communication, which may play an important role in public health surveillance in the future.

Social media provide a massive platform for patients to share information, discuss treatment, and promote mutual support; therefore, it may be a valuable database for disease research. Previous studies have used social media data to predict contagious diseases such as Acquired Immune Deficiency Syndrome (AIDS), syphilis, and flu [37–40]. Our findings indicated that social media data was able to reflect patients’ needs and concerns. According to the classification results, Baidu glaucoma bar users mainly sought professional advice related to symptoms or examination, accounting for 69.50% of posts seeking medical advice, and was much larger than that of seeking drug-related posts (13.89%) and surgery-related posts (16.63%). In addition, patients paid more attention to “whether they had glaucoma” and “whether it had progression”. However, Freia McGregor et al. analyzed five websites and found that people concentrated more on eye drops (37%), surgery (44%), and complementary therapies (11%) [22]. These differences hinted that, in China, doctors had ignored the cognition differences between doctors and patients about glaucoma [41]. Therefore, it is significant to pay attention to the effectiveness of doctor-patient communication and the popularization of medical knowledge.

Word cloud analysis is the visualization of text data; the more frequently a word appears, the larger area it occupies. In our study, the word cloud analysis showed that patients’ activities on social media were mainly focused on asking for help to interpret examination results, seeking or sharing medical experiences, and seeking better medical resources, which was consistent with the classification results. In addition, social media provides a space for patients to raise questions, share knowledge, and provide advice and support to each other. And the words “wardmate” and “friend” appeared in word cloud analysis, which reflected that newly diagnosed glaucoma patients tended to obtain emotional comfort through mutual communication to better psychological acceptance. Totally, “IOP”, “VF” and “examination” were the top three frequent words. As we all know, glaucoma is a group of diseases with characteristic optic nerve damage and VF defects, and IOP and VF examination are important factors in the diagnosis and prognosis. In addition, “blurred vision” was the secondary common symptom, and it was more frequent than “headache” and “eye distension”, which indicated that patients paid more attention to changes in visual function.

There are several kinds of treatment for glaucoma, including pharmacotherapy and surgical treatment [42–49]. We found that the most frequent drug name was “travoprost”, probably the most commonly used eye drop in China. Travoprost, a kind of prostaglandin, was widely used in glaucoma patients, and it has been confirmed to have prominent effects and fewer side-effect than other kinds of drugs [50, 51]. The above results showed that patients preferred treatment with fewer side effects and complications.

Word cloud analysis showed that negative emotion, “worry”, “fear”, and “discomfort”, was more frequent than optimistic emotion. Disease and treatment could significantly impact a patient’s life quality. Studies have shown that apparent anxiety will appear as soon as they are diagnosed with glaucoma, even without evident VF damage. It constantly negatively influences patients’ quality of life and well-being. Both ocular surface diseases caused by preservatives in eye drops [52–54] and ocular symptoms after surgery could negatively affect patients’ life quality and reduce patients’ compliance to treatment.

Many studies have combined social media with NLP to solve public health problems. Similarly, Albert Park et al. tracked health-related discussions on Reddit using NLP to classify the topic and purpose of the discussion and found that Reddit users were most concerned about the “risks” and “symptoms” of Ebola. In our study, both models achieved promising performance for detecting posts seeking medical advice, indicating that NLP could perform well in Chinese text and may be applied to large-scale Internet data.

Our study truly has some limitations. First, considering protecting users’ privacy, the website hid users’ addresses, so we could not obtain the general regional information, which restricted us from comparing the regional differences. Secondly, Baidu Tieba users were not the same as users on other online platforms, which may cause a bias in our results. Thirdly, due to the large difference in training data distribution, we cannot classify the training data in a more detailed way. In addition, when processing the training data, we took the whole content of a topic post as a sample, including the title, post content, and comments, which may cause interference in the model training.

Despite limitations, our study was the first attempt to use data from Baidu Tieba for analysis. Our preliminary results indicated that Chinese social media data could reflect patients’ focus on diseases, find priorities in doctor-patient communication, and cognitive differences, and NLP can classify large amounts of Chinese social media text data to quickly find patients’ priorities, and clinicians could capture information in a timely and efficient manner from social media platforms and provide more percise guidance for patients, which will provide a mutually supportive environment for airing questions, opinions and suggestions and improve communication efficiency, and NLP can classify large amounts of Chinese social media text data to quickly find patients’ priorities. In the long term, public health or clinical practitioners may try to carry out online medical services to better serve patients.

## Data Availability

The datasets used and analyzed in the present study are available from the corresponding author upon reasonable request.
